# USP18 Promotes Cholesterol Efflux and Mitigates Atherosclerosis by Deubiquitinating ABCG1

**DOI:** 10.1111/jcmm.70320

**Published:** 2025-01-13

**Authors:** Yang An, Chuxian Guo, Xiaoli Wang, Jiangjin Liu, Zhu Li, Jiuyang Ding, Qiaojun Zhang, Hongmei Zhou, Bing Xia, Jiawen Wang, Yanni Yu, Changwu Wan, Jie Wang, Jialin Dai

**Affiliations:** ^1^ School of Forensic Medicine Guizhou Medical University Guiyang China; ^2^ Department of Cardiology The First Affiliated Hospital of Jinan University Guangzhou China

**Keywords:** ABCG1, atherosclerosis, deubiquitinating enzyme, macrophages, USP18

## Abstract

Deubiquitinating enzymes (DUBs) are integral regulators of protein stability. Among these, Ubiquitin‐specific protease 18 (USP18) has emerged as a potential therapeutic target for heart failure. However, its precise role in atherosclerosis remains to be comprehensively understood. This study endeavours to examine the impact of USP18 on atherosclerosis and elucidate its corresponding molecular mechanisms. Our studies indicate an elevated expression of USP18 in human coronary atherosclerotic plaques. Notably, the knockdown of USP18 significantly exacerbated lipid accumulation in macrophages. This knockdown effect impaired cholesterol efflux and influenced the downregulation of ATP‐binding cassette transporter G1 (ABCG1) expression, achieved by altering the ubiquitination level of ABCG1. Comprehensive mechanistic studies unveiled that USP18 directly affiliates with ABCG1, reducing its ubiquitination and consequently bolstering ABCG1 stability within macrophages. Furthermore, in vivo studies elucidated that the knockdown of USP18 notably elevated atherosclerotic lesions and diminished ABCG1 levels in the plaques of Apoe^−/−^ mice. In summary, our results suggested that USP18 plays a crucial role in managing the progression of atherosclerosis by strengthening the expression of ABCG1 protein through its deubiquitinating effect on ABCG1.

## 1 Introduction

Atherosclerosis, a chronic arterial disease with significant global mortality [1], is typified by the accumulation of lipids and the infiltration of inflammatory cells within the arterial wall [2]. The formation of foam cells, primarily derived from macrophages [3], marks the distinctive characteristic of early atherosclerosis [4]. Hence, gaining a comprehensive understanding of the mechanism underlying foam cell formation might pave the way for identifying new therapeutic targets for atherosclerosis. In the event of a disorder causing disruption in macrophages' cholesterol metabolism haemostasis, an increase in the intake or a decrease in the outflow of oxidised low‐density lipoprotein (ox‐LDL) can lead to the transformation of macrophages into lipid‐rich foam cells [5, 6]. Macrophages uptake extracellularly modified LDL through receptor‐mediated phagocytosis and pinocytosis, a process associated with dysregulation of ox‐LDL uptake mediated by CD36 and the class A1 scavenger receptor (SR‐A1) [7, 8, 9]. The first and rate‐limiting step of reverse cholesterol transport (RCT), cholesterol efflux from macrophages to apolipoprotein AI (apoA‐I) or high‐density lipoprotein (HDL), is predominantly facilitated by ABC transporters, including ABCA1 and ABCG1 [5, 10, 11]. Research has demonstrated that ABCG1 facilitates the evacuation of surplus cholesterol from macrophages and orchestrates the establishment of HDLs, thereby transporting cholesterol to the liver for excretion [12]. In addition, ABCG1 stimulates cholesterol efflux [13]. Inhibition of ABCG1 results in intracellular cholesterol accumulation, demonstrating the critical role of ABCG1‐dependent cholesterol transport in lipid homeostasis and in stifling the progression of cardiovascular disease [11]. Hence, scrutinising the mechanisms governing ABCG1 expression could hold significance for devising a novel therapeutic strategy for atherosclerosis.

The ubiquitin‐proteasome system (UPS), accountable for the regulation of degradation in approximately 80% of proteins within eukaryotic cells, is a proteasome system prevalent throughout the human body [14]. Deubiquitinating enzymes (DUBs) potentially stabilise these proteins by inducing their deubiquitination [15]. Autopsy and immunohistochemical analysis performed by Hermann et al. on coronary arteries from patients suffering from acute coronary syndrome revealed an enhanced ubiquitin immunoreactivity within the unstable plaque regions [16]. Ubiquitin also showed an accumulated presence in the carotid plaques, which was associated with oxidative stress and proteasome activity dysfunction [17]. These findings suggest the potential critical role of DUBs in atherosclerosis development. Recent studies have further emphasised the essential role of USP18 within the cardiovascular system.

USP18, a member of the DUBs family, features prominently in regulating various cellular activities such as tumour metastasis, hepatic steatosis, and innate immune regulation [18, 19, 20, 21]. Recent studies have illustrated that USP18 can inhibit cardiac remodelling and arrest the development of heart failure through the TAK1‐p38/JNK1/2 signalling pathway [22]. Furthermore, USP18 has been detected at high levels within the serum of male hypertensive patients [23], suggesting a possible critical role in the initiation and progression of cardiovascular diseases. A different study has also indicated that ABCG1, degraded by UPS [24], potentially functions as a bridge in USP18‐regulated lipid metabolism during the development of atherosclerosis.

This study demonstrated a noticeable increase in USP18 within the human atherosclerotic plaque samples. Suppressing USP18 was found to obstruct cholesterol efflux in macrophages, which then triggers an accumulation of cellular lipids. Mechanistically, the repression of USP18 triggers a surge in the ubiquitination of ABCG1 and facilitates its degradation. To conclude, our findings indicate the protective influence of USP18 on atherosclerosis as well as delineate its possible mechanism, suggesting that USP18 can potentially serve as a therapeutic target for atherosclerosis.

## 2 Materials and Methods

### 2.1 Bioinformatics Analysis

The gene expression profiles for Familial Hypercholesterolemia (FH, GSE6054) were retrieved from the Gene Expression Omnibus (GEO) in the National Centre for Biotechnology Information (NCBI) database (http://www.ncbi.nlm.nih.gov/geo/). analysis of differentially expressed genes was conducted using the limma package in R (4.1.3 version); only genes with a false discovery rate of less than 0.05 and fold‐change equal to or greater than 1 were considered for further network construction. The heatmap and volcano plots of differentially expressed genes were produced using the SangerBox software (3.0 version), a complimentary online platform for data analysis [25].

### 2.2 Human Coronary Artery Samples

In the present study, subjects with Coronary Heart Disease (CHD, *n* = 10) and those without CHD (*n* = 10) were chosen to evaluate the expression levels of pertinent proteins in the left anterior descending branches. The CHD inclusion criteria included the formation of atheromatous plaque and a lumen stenosis of ≥ 50% in the anterior descending branch of the left coronary artery, coupled with death within 24 h, excluding incidents of violent death and unrelated terminal diseases. The explicit exclusion specifications were: (1) any time of death extending beyond 24 h (from the point of death to autopsy); (2) the deceased's affliction with alternative types of heart disorders such as dilated cardiomyopathy, Kashan disease, myocarditis, hypothyroid heart disease, constrictive pericarditis and heart amyloidosis; and (3) incidences of drug misuse. For the control group, the inclusion criteria encompassed accidental causes of death such as falls, traffic accidents and electric shocks, et al. It also required a pathology examination revealing an absence of heart disease or coronary stenosis and death within 24 h. The exclusion criteria for the control group paralleled those established for the CHD group [26]. The collection of all coronary artery samples used in this study occurred from January 2020 through December 2021, secured from the Forensic Judicial Expertise Centre at Guizhou Medical University. The study was administered in alignment with the Helsinki Declaration. Despite being unable to obtain informed consent statements from the majority of patients, due to either loss to follow‐up or death, the Ethics Committee of Guizhou Medical University sanctioned this study (approval number: 2022–278). It is worth noting that the origin of our research samples is derived from forensic autopsies instead of hospitals. Consequently, it is impossible for us to share the patients' clinical data with hospitals. Moreover, a portion of patients exhibited no signs or symptoms of coronary artery disease prior to their demise and had not been hospitalised. They succumbed to incidents like traffic accidents or falls from heights. Although coronary artery disease was identified during the autopsy, comprehensive clinical test results, including triglycerides, total cholesterol, LDL, and HDL, could not be obtained owing to the absence of relevant hospitalisation records.

### 2.3 Animals

Male homozygous Apoe^−/−^ mice of 6–8 weeks of age were procured from Beijing Sibeifu Biotechnology Co. Ltd. in China. Experiments on these animals were sanctioned by the Animal Ethics Committee of Guizhou Medical University, under the approval ID 2201619. The mice were randomly segregated into three groups, each with a subject size of ten. In the adenovirus group, mice were exposed to adenovirus‐USP18‐RNAi (Ad‐USP18, 1 × 10^10^ PFU/mouse) via tail‐vein injection. The control adenovirus group underwent a similar procedure, but were injected with control adenovirus‐hU6‐MCS‐CMV‐EGFP (Ad‐NC, 1 × 10^10^ PFU/mouse). Tail‐vein injections were done weekly for both groups. The mice in the high‐fat diet (HFD) group received no such treatment. All groups were kept on a free‐access HFD for 16 weeks, after which the mice were anaesthetised and sacrificed for tissue collection from the aorta, heart, and other organs.

### 2.4 Cell Lines and Culture Conditions

THP‐1 monocytes, procured from the Cell Bank of the Chinese Academy of Sciences (Shanghai, China), were cultured using RPMI 1640 medium (KeyGEN, China). This medium was supplemented with 12% Foetal Bovine Serum (FBS, Absin, China). The culture was maintained in a humidified incubator at 37°C supplemented with 5% CO_2_.

### 2.5 siRNA Transfection

The THP‐1 monocytes were cultured in plates, and treated with Phorbol 12‐myristate 13‐acetate (PMA, 100 ng/mL) medium to stimulate THP‐1 cell differentiation. After 24 h incubation, the medium was replaced. Subsequently, the THP‐1‐derived macrophages were transfected with either USP18‐specific siRNA or control siRNA (50 mM) using the siRNA‐reagent complex. The medium was refreshed after 48 h of the transfection, followed by relevant experiments.

### 2.6 Oil Red O Staining

The THP‐1 cells were seeded onto slides and exposed to USP18 siRNA or control siRNA for 48 h, followed by a 24‐h incubation with ox‐LDL (50 μg/mL) to prompt foam cell formation. Subsequent to this, the cells were rinsed with PBS and solidified with a 4% paraformaldehyde solution for 15 min. Thereafter, the cells underwent a 5‐min incubation with isopropanol, staining with 0.5% Oil Red O for 30 min, and a wash with 85% isopropanol before being stained with haematoxylin for counterstaining. Image capture was facilitated using the Motic digital slide scanner (EasyScan NFC 60), and quantitative analysis was executed with the aid of Image J software.

### 2.7 Immunofluorescence Staining

Paraffin sections underwent a decaraffinization process in xylene, a subsequent rehydration in gradient alcohols, and a final wash with PBS. Antigen repair was facilitated by the use of EDTA. As part of the protocol, these cells were sited onto a chamber slide and subjected to the prior treatment. PBS application was purposed for washing the treated cells, followed by the addition of 4% paraformaldehyde on the slide for a period of 15 min. Treatment of cells with 0.1% Triton X‐100 persisted for 10 min and was followed by blockage of cells with goat serum lasting 30 min at room temperature. Slides were then refrigerated at 4°C and left to incubate in the primary antibody solution throughout the night. After conducting a triple wash with PBS, the cells or tissues were incubated with either Alexa Fluor 488 or Alexa Fluor 594 conjugated secondary antibodies; this was done over a period of 1 h at room temperature. Lastly, slides were covered using DAPI, and images were procured using a Zeiss upright fluorescent microscope.

### 2.8 Multiplex Immunohistochemistry (mIHC) Staining

The Tyramide signal amplification technique was employed to conduct polychromatic immunofluorescence staining using a four‐colour multiple fluorescent immunohistochemical staining kit (Absin, abs50012). Paraffin sections were dewaxed in xylene and rehydrated by way of an alcohol gradient. Following this, antigen repair was completed using a microwave and sodium citrate buffer at a pH level of 6.0, before allowing them to cool to room temperature. Slides were incubated in primary antibody solution at 4°C overnight, subsequent to a 10‐min room temperature blocking in goat serum. Following three PBST washes, the slides were then exposed to their corresponding secondary antibodies for an hour. This was followed by a 10‐min incubation period with a fluorescent dye, which had been diluted using an amplification reagent. After this microwave repair process, the slides were allowed to cool to room temperature before another hour‐long incubation with secondary antibodies and an additional 10‐min period with diluted fluorescent dye. The process of multiple fluorescent staining required repetition of the previously detailed steps until all antigens were stained. Lastly, incubation with DAPI for 10 min concluded the process, after which the sections were sealed with an anti‐fluorescence quencher.

### 2.9 Cholesterol Efflux Assay

The THP‐1 monocytes were cultured in plates and subjected to treatment with either USP18‐specific siRNA or control siRNA for a period of 48 h. Thereafter, the medium was substituted with a fresh one devoid of phenol red. Following the treatment, THP‐1 macrophages were incubated with ox‐LDL (50 μg/mL) and 22‐[(7‐Nitrobenz‐2‐oxa‐1,3‐diazol‐4‐yl) amino]‐23,24‐dinor‐5‐cholen‐3β‐ol, also known as NBD‐Cholesterol, at a concentration of 5 μmol/L at a temperature of 37°C for the duration of 24 h. Subsequently, the initial medium was replaced by the RPMI medium containing HDL (50 μg/mL), and this composition was left to incubate at 37°C for another 12 h. Post incubation, the cellular culture medium and cell lysate were collected for analysis. The fluorescence intensity in both the cell culture medium and the cells themselves was measured using a multi‐functional enzyme labeler. The percentage of cholesterol efflux was calculated with the following formula: (medium count/(medium count + lysate count)) × 100%.

### 2.10 PCR Analysis

Total RNA was extracted from cells using the Trizol reagent (Vazyme, USA). The qRT‐PCR was executed utilising the SYBRSelect Master Mix (Vazyme, USA) on the Applied Biosystems 7500 Fast Real‐Time PCR System (Thermo Fisher Scientific, USA). The selected primers used are subsequently listed.

ABCG1: F:5′‐GCCATGAATGCCAGCAGTTACTC‐3′;

and R:5′‐TTCAGCAG GTCCGTCTCAGTG‐3′.

β‐actin: F:5′‐CATCATGAAGTGTGACGTGG‐3′;

and R:5′‐TCGTCATACTCCTGCTTGCT‐3′.

### 2.11 Western Blotting Assay

The cells or tissues were lysed using pre‐cooled RIPA buffer for a duration of 20 min, with a presence of protease inhibitors. Supernatants were subsequently collected through centrifugation at 12,000 rpm for 10 min at 4°C. Aliquots of the samples were subjected to SDS‐PAGE and transferred onto polyvinylidene difluoride (PVDF) membranes from Millipore Corporation, USA. The PVDF membranes were blocked using western blot high‐efficiency blocking solution from Gene First, Shanghai, China, for 10 min, and then incubated with primary antibodies. The antibodies used were against USP18 (1:1000; GB111923), ABCG1 (1:800; AP13578), ABCA1 (1:1000; PU115342), CD36 (1:1000; abs172281), SR‐A1 (1:1000; AP 24655), GAPDH (1:5000; GB15002), and ubiquitin (1:1000; M026378) at 4°C overnight. Following this, the membranes were incubated with HRP‐conjugated secondary antibodies (1:5000) for 1 h at room temperature. Immunoreactive bands were visualised using the ECL Plus kit, and GAPDH served as an internal control. Quantitative analysis of these bands was performed using Image J software.

### 2.12 Co‐Immunoprecipitation (Co‐IP) and In Vivo Ubiquitination Assay

According to the Co‐IP kit producer's guidelines (Abmart, Shanghai, China), THP‐1‐derived macrophages were collected and washed with ice‐cold PBS before a 10‐min lysis using a buffer containing a cocktail proteinase inhibitor was performed. The lysed cells were scraped off the culture dish and transferred into a microcentrifuge tube. Subsequently, by centrifugation (12,000 rpm for 10 min at 4°C), the supernatant was moved to a new microcentrifuge tube. The cell lysates were then incubated with a specific primary antibody (USP18 or ABCG1) for 2 h at 4°C, followed by an overnight incubation with Protein A/G magnetic beads on a rocker platform at 4°C. The co‐precipitated proteins were washed thrice with wash buffer. Lastly, the co‐precipitated were reconstituted in 200 μL 1 * SDS sample buffer, and the sample was boiled for 10 min. The magnetic beads were secured to a magnetic rack, and the immunoprecipitates were subjected to SDS‐PAGE. In the in vivo ubiquitination assay, macrophage cells were treated with either USP18‐specific siRNA or control siRNA for 48 h. Then, the cell lysate was immunoprecipitated with an anti‐ABCG1 antibody, and an anti‐Ub antibody assessed the ubiquitination level of ABCG1.

### 2.13 Molecular Docking

The docking analysis was performed using “H‐DOCK” with its default parameters (http://hdock.phys.hust.edu.cn/). The structural models for USP18 (Uniprot ID: Q9UMW8) and ABCG1 (Uniprot ID: P45844) were acquired from the predictions of the structural database AlphaFold (https://alphafold.com/). The prediction of protein–protein interaction was accomplished through H‐DOCK, with the final free energy being determined from the docking outcomes in PDBePISA (https://www.ebi.ac.uk/msd‐srv/prot_int/pistart.html). The interaction model was chosen, and the analysis and plotting of the interaction sites were carried out based on the estimate of optimal free energy.

### 2.14 Data Analysis

Statistical analyses were conducted using GraphPad Prism (version 9.5.1) software. The results are presented as the mean ± SEM. For two‐group comparisons, the unpaired two‐tailed Student's *t*‐test was employed. In contrast, one‐way or two‐way ANOVA, followed by the Tukey's post hoc test, was utilised for comparisons involving more than two groups. A *p*‐value < 0.05 was considered to be indicative of statistical significance.

## 3 Result

### 3.1 Increased USP18 Expression in Atherosclerotic Plaques

**FIGURE 1:**
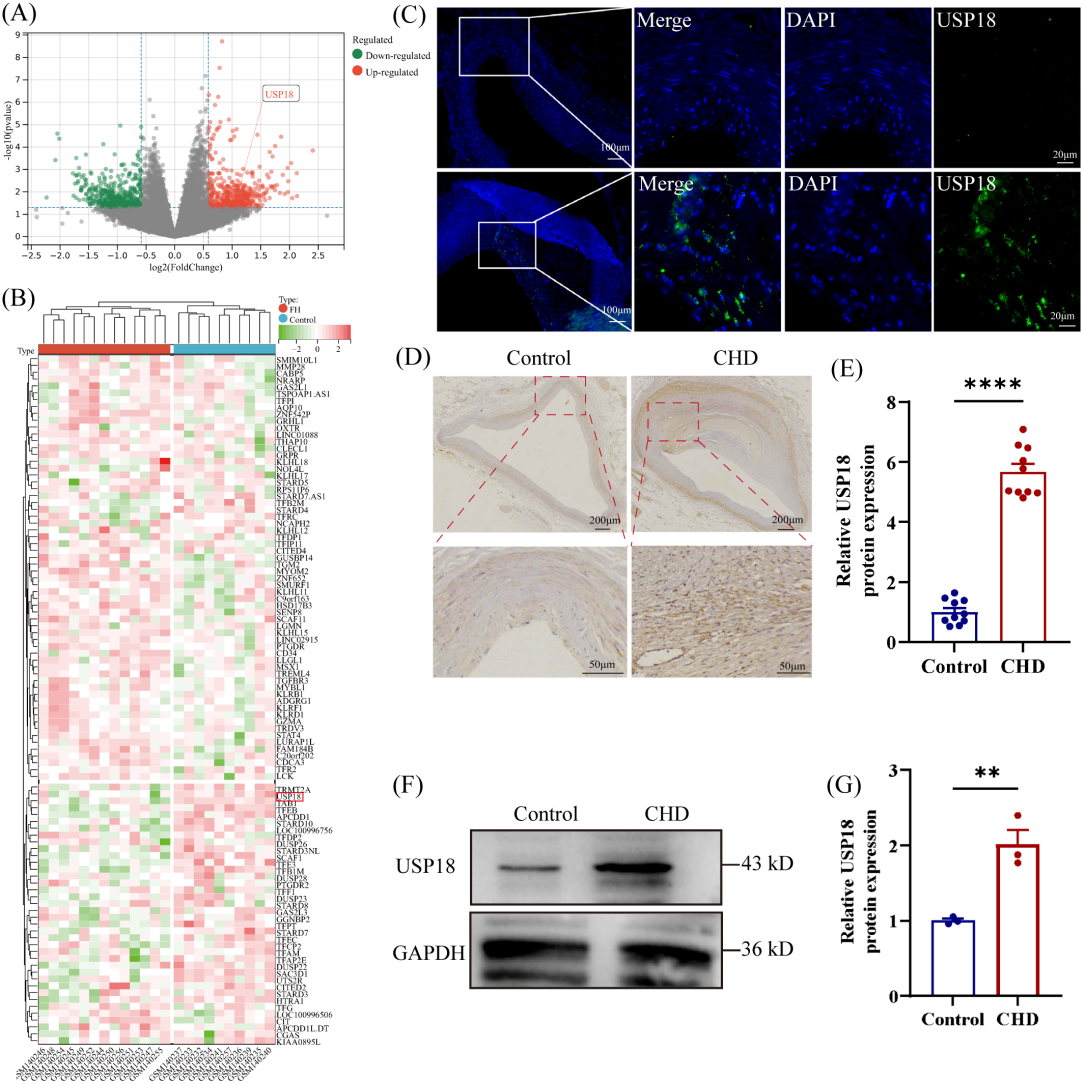
USP18 is upregulated during the formation of atherosclerosis. (A, B) The expression levels of USP18 in Familial Hypercholesterolemia (GSE6054) were quantified utilising microarray analysis and volcano plot drawn from GEO datasets (red signifying up‐regulation and green illustrating down‐regulation). (C) Immunofluorescence staining was performed to detect USP18 in these plaques. (D, E) Immunohistochemical staining was utilised to assess USP18 levels in coronary atherosclerotic plaques, with the mean optical density of USP18 staining measured (*n* = 10). (F, G) Western Blot analysis of USP18 protein levels in coronary atherosclerotic plaques (*n* = 3). **p < 0.01, ****p < 0.0001.

Familial Hypercholesterolemia (FH) is a significant risk factor for Atherosclerotic Cardiovascular Disease (ASCVD) [27]. In an attempt to identify differentially expressed genes in FH, we explored the GSE6054 dataset derived from the GEO database. Our analysis revealed that patients with FH exhibit a notably higher level of USP18 expression compared to those without hypercholesterolemia as illustrated by the heatmap and volcano plot (Figure 1A,B). Further measurement of USP18 expression in human coronary arteries manifested through Immunofluorescence showed an increased and widespread presence in atherosclerotic plaques (Figure 1C). This finding was substantiated by results obtained from immunohistochemical staining (Figure 1D,E). Moreover, consistent with these findings, the Western blot analysis demonstrated an elevated level of USP18 in coronary atherosclerotic plaques (Figure 1F,G). Taken collectively, these results suggest a correlation between USP18 and the progression of atherosclerosis.

### 3.2 USP18 Knockdown Accelerates the Development of Atherosclerosis in Apoe^−/−^ Mice

**FIGURE 2:**
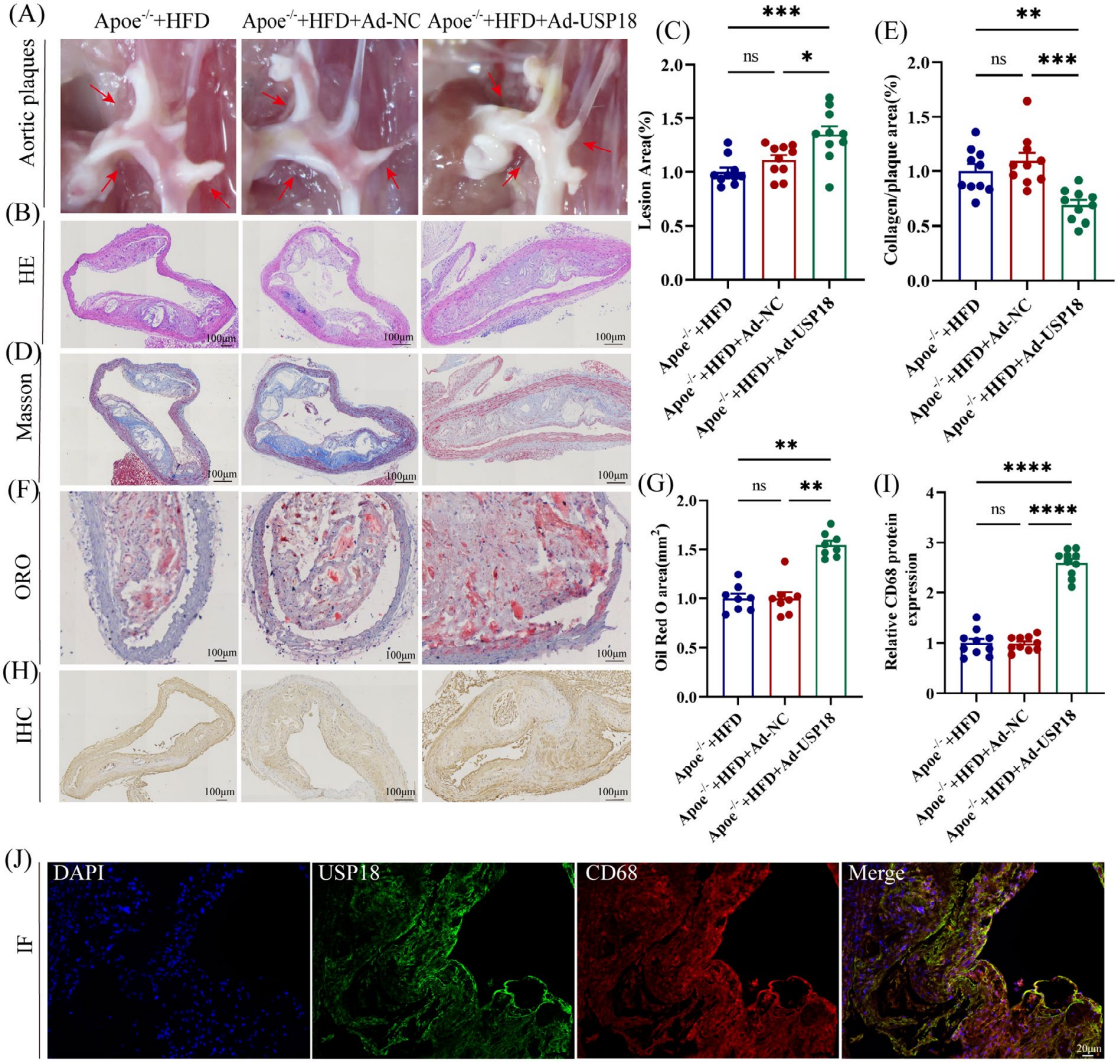
USP18 Knockdown Increases Atherosclerotic Lesion Area in Apoe^−/−^ Mice. Apoe^−/−^ mice were intravenously administered with Adenovirus‐USP18‐RNAi (Ad‐USP18) or control adenovirus (Ad‐NC) through the tail vein. The mice in the High‐Fat Diet (HFD) group, however, were not treated. All the mice were subjected to a high‐fat diet for a period of 16 weeks with each group containing ten animals. (A) Representative images of white plaque at the aortic arch site. (B) HE staining of aortic section (*n* = 10). (C) Quantification of aortic plaque area. (D) Representative images showing Masson's trichrome staining of aortic cross‐sections (*n* = 10). (E) Quantification of collagen fibre content. (F) Oil red O staining of aortic sections (*n* = 8). (G) Quantification of lipid content in plaques. (H) Immunohistochemical analysis of CD68 macrophages (*n* = 10). (I) Quantification of CD68‐positive areas within plaques. (J) Immunofluorescence staining for USP18 (green) and CD68 (red) in aorta (*n* = 3). *p < 0.05, **p < 0.01, ***p < 0.001, ****p < 0.0001.

The exploration of USP18's role in atherosclerotic plaque formation was conducted in vivo. Apoe^−/−^ mice were injected with either adenovirus‐USP18‐RNAi (Ad‐USP18) or hU6‐MCS‐CMV‐EGFP (Ad‐NC), while the High Fat Diet (HFD) group remained untreated. All three groups were then subject to a HFD for 16 weeks. We observed an increase in aortic atherosclerotic lesion areas in Apoe^−/−^ mice with USP18 knockdown, compared to the HFD and Ad‐NC groups, as shown in Figure 2A. In addition, examination of HE, Oil Red O, and Masson's trichrome‐stained aortic cross‐sections revealed a significant increase in lesion areas in USP18 knockdown Apoe^−/−^ mice (Figure 2B,G). Further investigations were also carried out on macrophage infiltration within the plaques. Immunohistochemical data suggested an increase in CD68‐positive cells within plaques due to USP18 knockdown (Figure 2H,I). Subsequently, we examined the expression of USP18 in macrophages. Immunofluorescence analysis confirmed co‐localisation of CD68 within plaques with USP18 (Figure 2J). These observations suggest USP18 may have a potential protective effect towards atherosclerosis.

**FIGURE 3:**
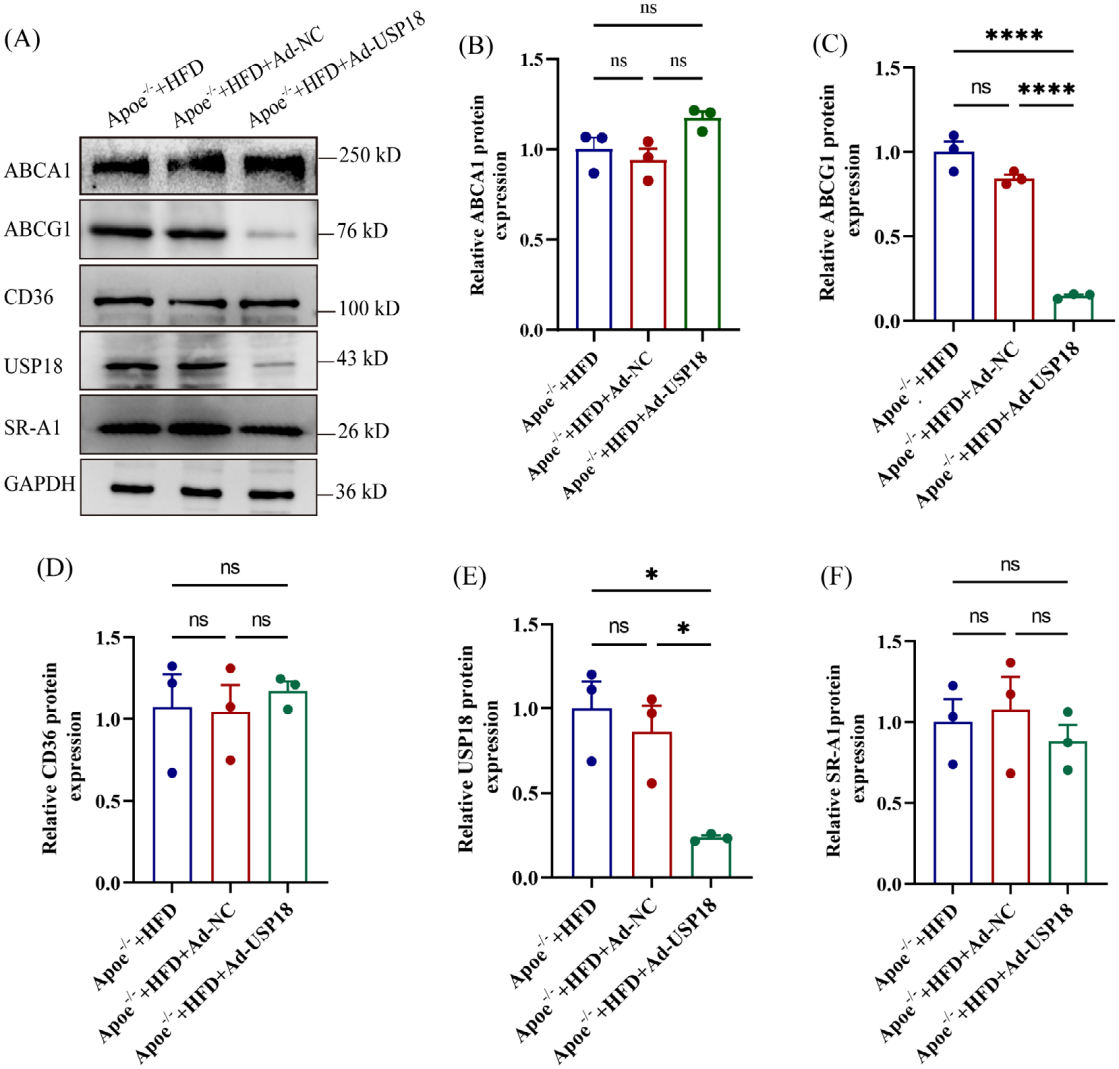
USP18 knockdown downregulates ABCG1 expression in Apoe^−/−^ mice. (A) Effect of USP18 on Expression of ABCA1, ABCG1, CD36, and SR‐A1 as Detected by Western blot (*n* = 3). (B–F) Quantification of Western blot data shown in (A). *p < 0.05, ****p < 0.0001.

Considering the pivotal role of metabolic imbalance in cholesterol as a constituent cause of atherosclerosis, we investigated the concentrations of proteins affiliated with cholesterol absorption and discharge, inclusive of CD36, SR‐A1, ABCA1, and ABCG1. Evidence from western blot findings suggests that lowering the levels of USP18 protein elicits a downregulation in the ABCG1 protein expression within the aorta of Apoe^−/−^ mice, whilst levels of CD36, SR‐A1, and ABCA1 proteins remained constant (observed in Figure 3A–F). This study thereby proposes that the progression of atherosclerosis may be modulated by USP18 through its regulation over ABCG1 expression.

### 3.3 USP18 Knockdown Promotes Lipid Accumulation in THP‐1‐Derived Macrophages

**FIGURE 4:**
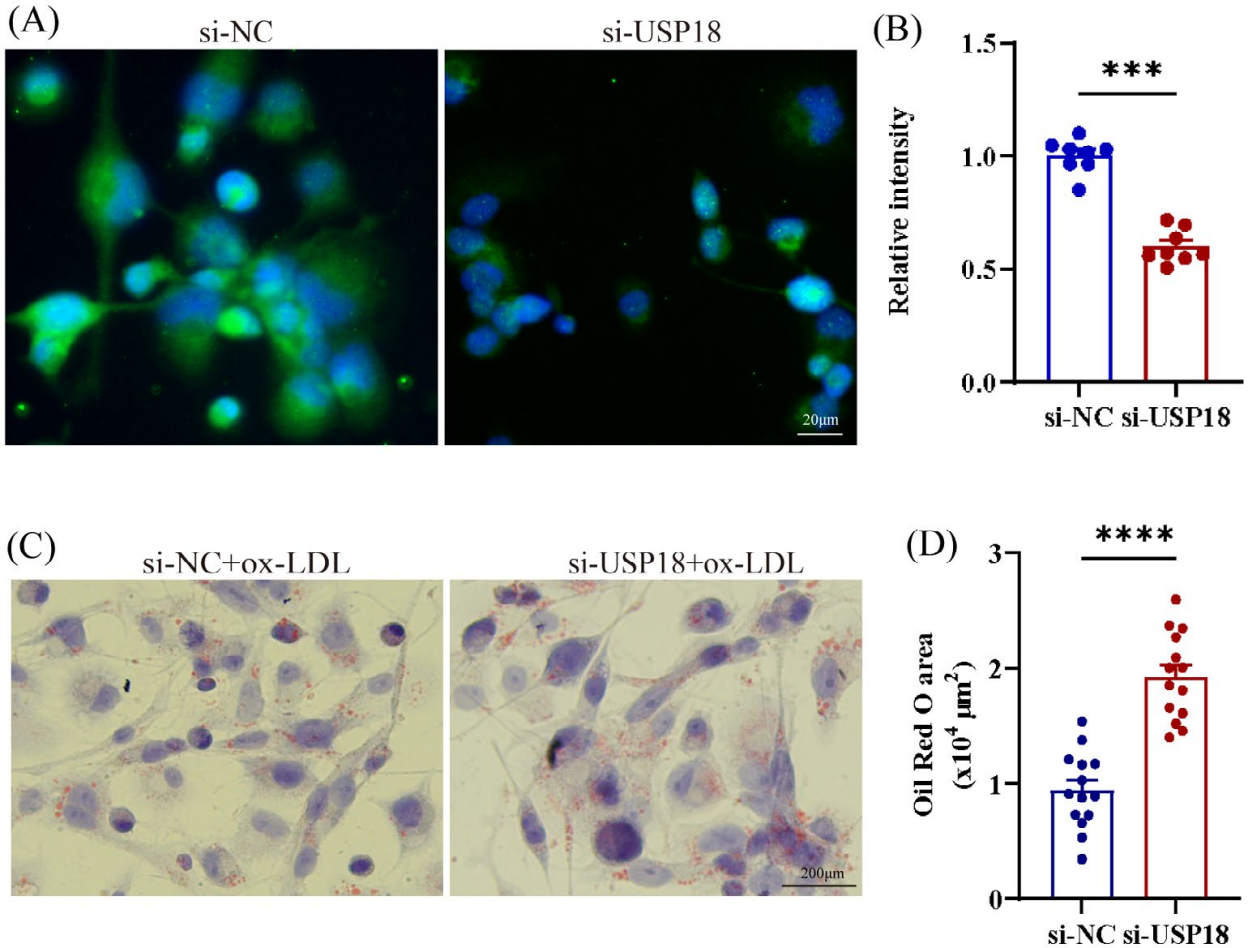
USP18 deficiency increases foam cell formation. (A, B) The macrophages derived from THP‐1 were treated with either USP18‐siRNA or control siRNA, the fluorescence intensity of these cells was ascertained via immunofluorescence (*n* = 8). (C, D) Lipid deposition in macrophages was assessed with oil red O staining (*n* = 14), and the Oil‐red O‐positive area was measured by Image J software. ***p < 0.001, ****p < 0.0001.

In this research, the effect of USP18 on foam cell formation was analysed, leveraging USP18‐siRNA to transfect THP‐1‐derived macrophages. A successful inhibition of USP18 was confirmed through immunofluorescence analysis (Figure 4A,B). Thereafter, foam cell formation was observed post‐exposure of these macrophages to ox‐LDL for a duration of 24 h. An intriguing escalation in intracellular oil red O particles became evident upon the inhibition of USP18, as illustrated by Oil red O staining (Figure 4C,D). This exposes that a downregulation of USP18 significantly escalates macrophage lipid accumulation.

### 3.4 USP18 Knockdown Hinders ABCG1‐Dependent Cholesterol Efflux

**FIGURE 5:**
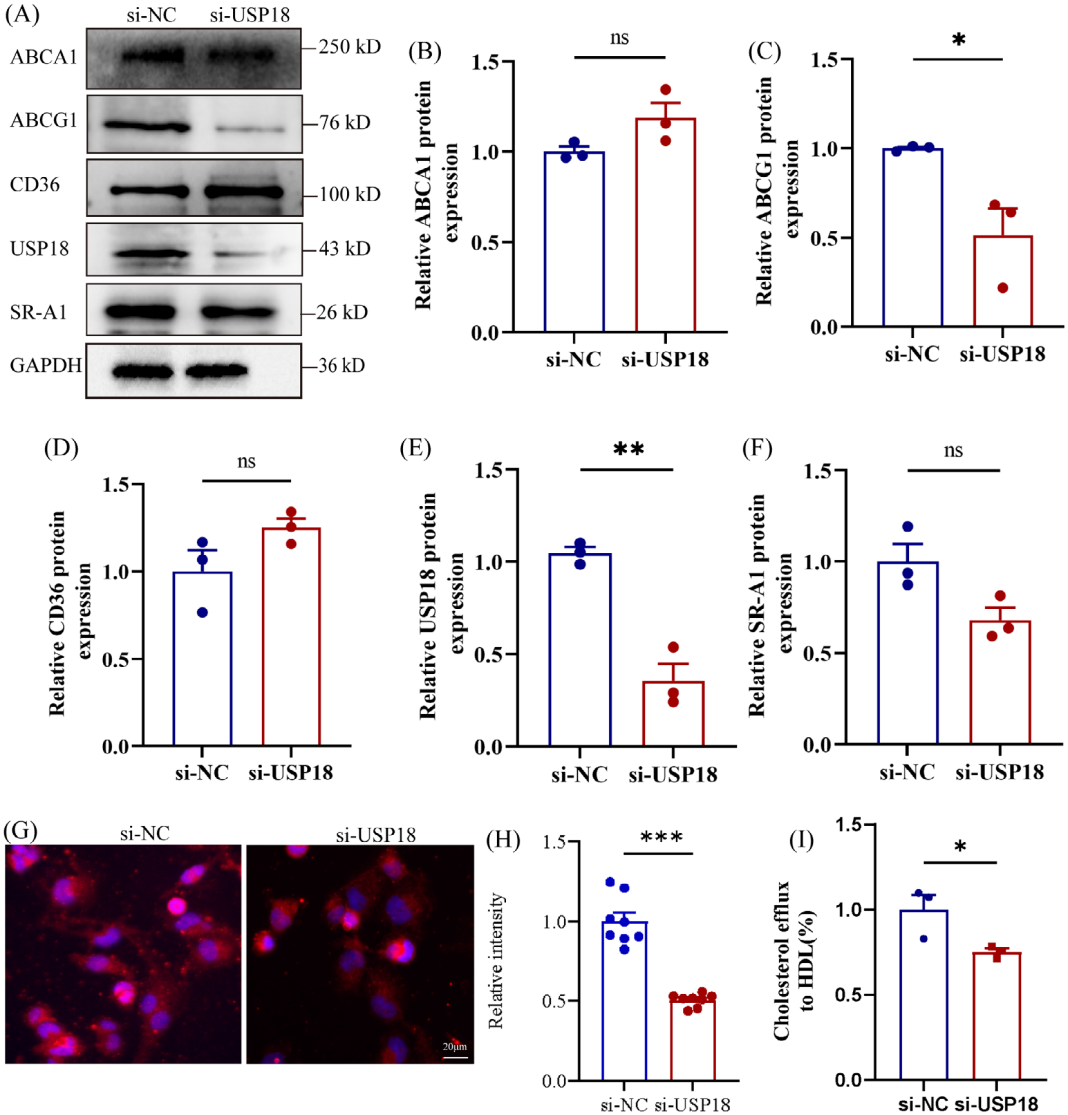
USP18 knockdown promotes the HDL‐mediated cholesterol efflux in macrophages. (A) The macrophages derived from THP‐1 were treated with either USP18‐siRNA or control siRNA, and the expression of ABCA1, ABCG1, CD36 and SR‐A1 was determined by Western blot (*n* = 3). (B‐F) Quantification of Western blot data shown in (A). (G, H) The macrophages derived from THP‐1 were treated with either USP18‐siRNA or control siRNA, and the fluorescence intensity of these cells was ascertained via immunofluorescence (*n* = 8). (I) Quantification of cholesterol efflux to HDL (*n* = 3). *p < 0.05, **p < 0.01, ***p < 0.001.

Foam cells, primarily derived from macrophages, form as a result of excessive lipid accumulation. This phenomenon often results from an imbalance between cholesterol influx and efflux [5, 28]. Critical receptors like CD36 and SR‐A1 mediate the cholesterol uptake pathway [29, 30], while essential proteins like ABCA1 and ABCG1 facilitate cholesterol efflux to ApoA‐I and HDL [31]. To investigate the influence of USP18 on scavenger receptors and ABC transporter proteins, we transfected THP‐1‐derived macrophages with USP18‐siRNA. We subsequently examined the protein levels of CD36, SR‐A1, ABCA1, and ABCG1. Western blot analysis demonstrated a decrease in ABCG1 protein expression after USP18 inhibition, whereas ABCA1, CD36, and SR‐A1 levels were unaffected (Figure 5A–F). Immunofluorescence results supported the diminution of ABCG1 expression upon USP18 inhibition (Figure 5E–H). Hence, further measurements were conducted to evaluate cholesterol efflux efficiency in macrophages. The results of the cholesterol efflux assay indicated a 26% decrease in cholesterol efflux to HDL in macrophages after silencing USP18, compared with the control group, as shown in Figure 5I. Accordingly, these results suggest that USP18 plays a role in foam cell formation, regulating ABCG1‐mediated cholesterol efflux.

### 3.5 USP18 Interacts With ABCG1 and Stabilises Its Expression

**FIGURE 6:**
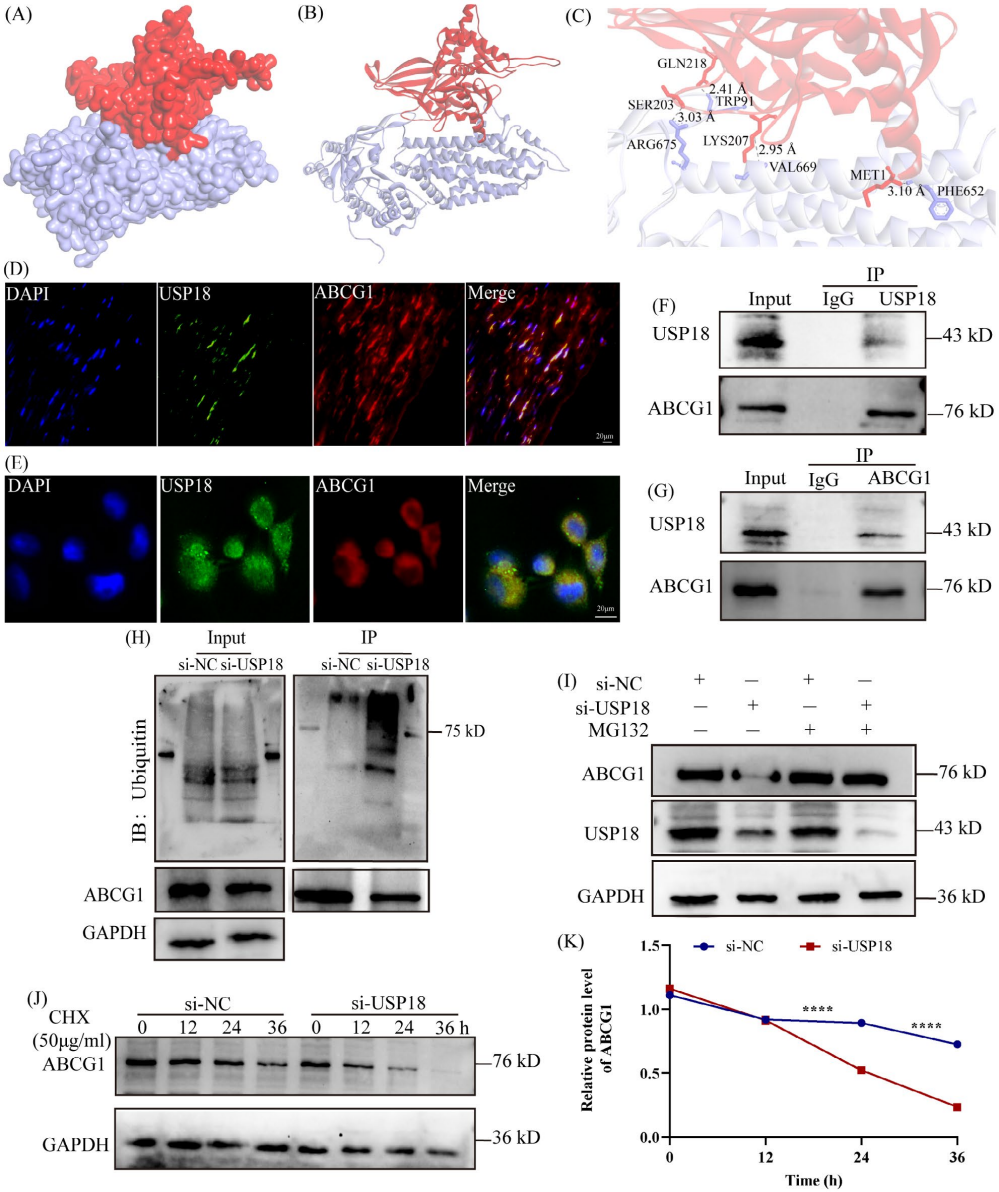
USP18 interacts with ABCG1 and stabilises ABCG1 expression through deubiquitination. (A, B) 3D crystal structures of USP18 (red) and ABCG1 (purple). (C) USP18 and ABCG1 interacting residues. (D) Immunofluorescence showing the co‐localization of USP18 (green) and ABCG1 (red) in human coronary atherosclerotic plaques (*n* = 3). (E) Immunofluorescence showing the co‐localization of USP18 (green) and ABCG1 (red) in macrophages (*n* = 3). (F) Protein samples were procured from macrophages derived from THP‐1 cells and then immunoprecipitated using beads coated with the USP18 antibody. Subsequent analysis was performed using Western blot tests for USP18 and ABCG1 proteins. (G) Immunoprecipitation was performed using ABCG1 antibody‐coated beads, followed by immunoblotting for the proteins USP18 and ABCG1. (H) Macrophages underwent treatment with either USP18‐siRNA or a control siRNA. ABCG1 antibody beads were immunoprecipitated. The ubiquitin (Ub) levels within ABCG1 were then detected through Western blot analysis. (I) Western blot was used to detect ABCG1 protein level in macrophages stably transfected with the control siRNA or the USP18‐siRNA in the present of MG132 (10 μmol/L). (J, K) Western blot was used to detect the protein degradation rate of ABCG1 in macrophages stably transfected with USP18‐siRNA or control siRNA in the presence of CHX (50 μg/mL). ****p < 0.0001.

Ogura et al. demonstrated that the UPS plays a crucial role in the degradation of ABCG1, which impacts reverse cholesterol transport (RCT) in vivo [24]. Given the function of USP18 as a DUB [18], it was hypothesised that USP18 might regulate the ubiquitination and subsequent degradation of ABCG1. To corroborate this hypothesis, molecular docking was employed to anticipate the potential interaction between USP18 and ABCG1. The 3D crystal structure analysis that ensued revealed a significant interaction, with a calculated free energy of −20 kcal/mol (a value below zero signifies significance), between USP18 and ABCG1, as depicted in Figure 6A–C and delineated in Table 1. It is important to note that this result is based solely on theoretical computational modelling, and its accuracy and validity in real biological systems require further confirmation. Therefore, the results of molecular docking should be considered as provisional conclusions with certain limitations. Immunofluorescence analysis continually demonstrated an intense co‐localisation of USP18 and ABCG1 in human coronary atherosclerotic plaques and THP‐1‐derived macrophages (Figure 6D,E). An interaction between USP18 and ABCG1 in THP‐1‐derived macrophage cells was further substantiated by subsequent co‐immunoprecipitation analysis using endogenous USP18 and ABCG1 antibodies, as evident in Figure 6F,G. Following the silencing of USP18, we undertook an investigation into the ubiquitin level of ABCG1 in cells. The Western blot analysis results revealed a pronounced increase in ABCG1 ubiquitin level in macrophages upon inhibition of USP18 (Figure 6H). Interestingly, macrophages exhibiting USP18 knockdown restored ABCG1 protein levels after treatment with the proteasome inhibitor MG132 (Figure 6I). This infers that USP18 may play a regulatory role in ABCG1 expression through the proteasome pathway. Additionally, we examined the degradation of ABCG1 protein in cells displaying USP18 knockdown, after treatment with cycloheximide (CHX, an inhibitor of all intracellular protein synthesis). The results indicated an enhanced ABCG1 protein degradation in macrophages when USP18 was suppressed (Figure 6J,K). Considering that ubiquitination is a post‐translational modification process, we evaluated the mRNA expression of ABCG1 alongside its protein levels. RT‐PCR results showed that USP18 inhibition had no effect on ABCG1 mRNA levels (Figure S1). This suggests that USP18 regulates ABCG1 protein level rather than its mRNA level. In essence, the evidence provided underscores USP18's role as a DUB instrumental in stabilising the expression of ABCG1 in macrophages via the ubiquitin‐proteasome pathway.

**TABLE 1:** Atoms involved in hydrogen bond formation and bond lengths of chemical bonds.

ABCG1	USP18	Distance [Å]	Category
TRP91	GLN218	2.41	Hydrogen bonds
VAL669	LYS207	2.95	Hydrogen bonds
ARG675	SER203	3.01	Hydrogen bonds
PHE652	MET1	3.10	Hydrogen bonds

## 4 Discussion

This study elucidates the pivotal role of USP18 in foam cell formation and atherosclerosis development. It is suggested from our data that an up‐regulation in the human coronary atherosclerotic plaque of USP18 takes place. Significantly, an impediment on ABCG1‐dependent cholesterol efflux and an enhancement of lipid accumulation in macrophages are observed with the knockdown of USP18. Furthermore, there is an acceleration in atherosclerotic plaque formation in Apoe^−/−^ mice upon the knockdown of USP18. Thus, the evidence provided by our data underlines the protective influence of USP18 in atherosclerosis development.

Atherosclerosis is a disease characterised by an accumulation of cholesterol and enduring inflammation within arterial walls [32]. Current studies have indicated that the formation of foam cells is chief to the development of atherosclerosis, with the main contributors being compromised cholesterol intake or output leading to foam cell and atherosclerosis formation [4, 33]. Preserving a dynamic balance of cell cholesterol metabolism is a crucial step in atherosclerosis prevention [6, 34]. Although USP18 has been identified as significant in lipid metabolism regulation [20], its specific involvement in atherosclerosis remains ambiguous. The study by An et al. demonstrated that total cholesterol (TC), triglycerides (TG), and lipid droplets in the livers of hepatocyte‐specific USP18 transgenic (USP18 HTG) mice on a high‐fat diet were reduced. Additionally, the mRNA expression levels of genes associated with cholesterol synthesis and uptake were decreased, whereas those related to cholesterol efflux were increased in these mice. Moreover, serum levels of TC, TG, and low‐density lipoprotein (LDL) were reduced, while HDL levels were elevated in USP18 HTG mice. These findings suggest that USP18 overexpression alleviates hepatocellular steatosis. Mechanistically, USP18 mitigates hepatic steatosis by interacting with and deubiquitinating TGFβ‐activated kinase 1 (TAK1), thereby inhibiting its activation and consequently suppressing the downstream JNK and NF‐κB signalling pathways [20]. This study investigates the USP18 expression in dysregulated cholesterol metabolism diseases, like FH, through the GSE6054 dataset to project its potential role in atherosclerosis [35]. FH—a predominately autosomal dominant disorder—is chiefly identified by elevated LDL cholesterol levels in plasma [36]. FH patients have been observed at an appreciably heightened risk of premature cardiovascular diseases and atherosclerosis [37]. They also tend to develop significant atherosclerotic plaques in coronary, carotid, thoracic, and abdominal arteries [38]. Our findings indicate an elevated USP18 expression in FH patients and even in human coronary atherosclerotic plaques, potentially due to a negative feedback system or compensatory failure. The study doesn't, however, identify a specific mechanism for this USP18 feedback or compensatory failure, which will be our subsequent study's focus. Significantly, we find that a reduction in USP18 escalates ox‐LDL‐induced foam cell formation in macrophages and enlarges the aortic atherosclerotic lesion area in Apoe^−/−^ mice. This aligns with previous findings that reduced ABCG1 expression in macrophages may augment atherosclerotic lesions due to cholesterol efflux impairment and subsequent foam cell formation enhancement [11, 34].

Our research initially verified the existing interaction between USP18 and ABCG1 through molecular docking (Figure 6A–C), with a calculated free energy of −20 kcal/mol. In the context of molecular docking, the free energy primarily evaluates the stability and feasibility of binding between proteins and ligands. The sign and magnitude of free energy reflect the nature and strength of intermolecular interactions. A negative free energy change (ΔG) indicates that the protein‐ligand binding process is spontaneous, reflecting a tendency of the ligand to bind to the protein. The larger the negative value of ΔG, the more stable the binding and the stronger the affinity. Conversely, a positive ΔG suggests that the binding is not likely to proceed spontaneously under the current conditions [39]. Furthermore, in vitro analyses substantiated the impediment of cholesterol efflux and promotion of foam cell formation when USP18 was silenced. Using these outcomes, we investigated the connection between USP18 and ABCG1, demonstrating that USP18's inhibition results in a decrease of ABCG1 protein expression. Concurrently, the ABCA1 protein expression remains unaffected by the silencing of USP18. The examination of the CD36 and SR‐A1 [7, 8] protein levels—the primary regulators of cholesterol intake—showed no significant alterations following USP18 inhibition. As such, we omitted the analysis of USP18's impact on cholesterol uptake. Collectively, the evidence suggests USP18's involvement in foam cell formation through the regulation of ABCG1 expression.

We subsequently delved into the molecular processes through which USP18 modulates ABCG1 protein expression. Data revealed that USP18 interacts with ABCG1 and curbs proteasome‐driven ABCG1 degradation via the removal of ubiquitin chains found in macrophages, thereby reinforcing ABCG1 expression within these cells. In a similar vein, USP18 was determined to bolster tumour proliferation, migration, and invasion by deubiquitinating ZEB1 and Snail1 and solidifying their expression [18, 19]. Moreover, a study by Ogura and associates established that ABCG1 degradation can occur through the UPS [24]. These findings support the notion that USP18 serves as a DUB for ABCG1. Further, it suggests that USP18 knockdown encourages foam cell formation and expedites atherosclerosis progression by enhancing ABCG1 degradation.

Notwithstanding, our study possesses several limitations. Firstly, we did not construct a distinctive macrophage knockout USP18 animal model, thereby restricting us from exploring the implications of macrophage‐specific USP18 on the evolution of atherosclerosis. Regrettably, due to a delay in separating the serum after collecting orbital blood from the animals, erythrocyte rupture occurred over time, resulting in hemolysis. Consequently, we were unable to assess the serum levels of cholesterol, triglycerides, LDL, and HDL in the animal model. Secondly, in light of the protein–protein docking results, we are unable to evaluate ubiquitination on protein–protein interaction. Finally, while we examined the proteasomal degradation pathway of ABCG1, we did not extend our investigation to other degradation pathways such as that by calcineurin of ABCG1.

## 5 Conclusions

In summarising our findings, we've presented evidence highlighting the beneficial role of USP18 in atherosclerosis. We discovered that USP18 functions as a DUB for ABCG1 within macrophages. Noteworthy is the interaction between USP18 and ABCG1, resulting in the reduction of ABCG1 polyubiquitination. This reaction promotes ABCG1 stability and promotes cholesterol efflux from macrophages. With this in view, our findings underscore the mechanism by which USP18 inhibits atherosclerosis development, signifying that USP18 targeting may serve as a potential strategy for atherosclerosis prevention and treatment.

## Author Contributions

**Jialin Dai:** funding acquisition (equal), validation (equal), writing – review and editing (equal). **Yang An:** conceptualization (equal), project administration (equal), writing – original draft (equal). **Xiaoli Wang**: methodology (equal) and formal analysis (equal). **Chuxian Guo:** conceptualization (equal), formal analysis (equal), software (equal). **Jiangjin Liu:** resources (equal). **Zhu Li:** resources (equal). **Jiuyang Ding:** validation (equal). **Qiaojun Zhang:** resources (equal). **Hongmei Zhou:** software (equal). **Bing Xia:** writing – review, funding acquisition (equal) and editing (equal). **Jiawen Wang:** writing – review and editing (equal). **Yanni Yu:** writing – review and editing (equal). **Changwu Wan:** writing – review and editing (equal). **Jie Wang:** writing – review and editing (equal).

## Conflicts of Interest

The authors declare no conflicts of interest.

## Supporting information


Figure S1

## Data Availability

The data that support the findings of this study are available from the corresponding author upon reasonable request.
