# Supplementary material for: USP18 Promotes Cholesterol Efflux and Mitigates Atherosclerosis by Deubiquitinating ABCG1
Source: J Cell Mol Med. 2025 Jan 13;29(1):e70320. doi: 10.1111/jcmm.70320 (PMC11728483; doi:10.1111/jcmm.70320)
Supplement: Supplementary file 1 — Figure S1 [file JCMM-29-e70320-s001.docx]

| 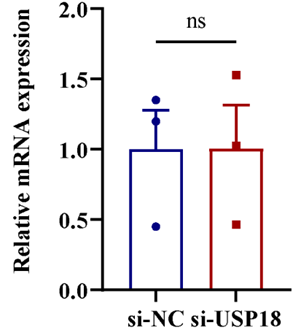 |
| --- |
| **Figure S1. The Influence of USP18 on ABCG1 mRNA expression levels.**  The total RNAs were extracted from macrophages that had been treated with USP18 siRNA for a specified duration, followed by a RT-qPCR analysis to determine ABCG1 mRNA expression levels. |
